# Mobility Classification of LoRaWAN Nodes Using Machine Learning at Network Level

**DOI:** 10.3390/s23041806

**Published:** 2023-02-06

**Authors:** Lorenzo Vangelista, Ivano Calabrese, Alessandro Cattapan

**Affiliations:** 1 Department of Information Engineering, University of Padova, Italy and Wireless and More srl, 35131 Padova, Italy; 2 A2ASmartCity, 25124 Brescia, Italy; 3 Wireless and More srl, 35131 Padova, Italy

**Keywords:** LPWAN, ADR, LoRaWAN

## Abstract

LoRaWAN networks rely heavily on the adaptive data rate algorithm to achieve good link reliability and to support the required density of end devices. However, to be effective the adaptive data rate algorithm needs to be tuned according to the level of mobility of each end device. For that purpose, different adaptive data rate algorithms have been developed for the different levels of mobility of end devices, e.g., for static or mobile end devices. In this paper, we describe and evaluate a new and effective method for determining the level of mobility of end devices based on machine learning techniques and specifically on the support vector machine supervised learning method. The proposed method does not rely on the location capability of LoRaWAN networks; instead, it relies only on data always available at the LoRaWAN network server. Moreover, the performance of this method in a real LoRaWAN network is assessed; the results give clear evidence of the effectiveness and reliability of the proposed machine learning approach.

## 1. Introduction

The low power wide area networks (LPWAN) paradigm is gaining a lot of momentum in the field of massive Internet of things (IoT) for its peculiarity of providing wide-area coverage while having low power requirements for transmission [1].

In this paper, we focus on LoRaWAN, the most prominent LPWAN technology working in the unlicensed spectrum. LoRaWAN Chapter 3 in Ref. [1] is characterized by a star topology, whereby the LoRa end devices (EDs) are connected to gateways (GWs) that act in principle as simple forwarders toward the network server (NS). The wireless communication between the EDs and the GWs takes place in the sub-GHz part of the unlicensed spectrum, which is quite limited. Efficient management of the very limited radio resources is thus essential to connect the very large amount of EDs that the LoRaWAN networks are expected to deal with in the massive IoT paradigm.

The LoRaWAN networks are often deployed in challenging radio environments in which the variability of the link quality is high due to various factors: obstacles, an urban scenario [2], ED mobility, and deep indoor environments [3].

A key point of the LoRaWAN technology is its ability to trade the data rate for coverage and vice versa, i.e., long-range communication can be established at the cost of a low data rate. The data rate and the power of the EDs are finely tuned by the adaptive data rate (ADR) algorithm running on LoRaWAN NS. In particular, the canonical LoRaWAN ADR [4] is not very efficient in the case of mobile nodes [5], and if the link conditions change or the network size increases too much, the convergence time of the ADR mechanism is quite high [6]. We quote the following from [4]:

For mobile EDs, the network-based ADR strategy does not work because of the unpredictable channel attenuation which occurs as the ED moves. Rather, with mobile EDs, ADR is performed “blindly” by the end device. This is referred to as “Blind ADR” [7].

In addition to [7], some strategies to solve this problem rely on a sort of network slicing method that appears in the literature, such as in [8,9], wherein the nodes’ transmission’s 40 parameters are tuned with respect to their mobility. The effectiveness of these approaches comes from their taking a proactive approach to the mobility of the EDs, in particular by monitoring the degree of mobility of an ED in which it is possible to update the transmission parameters to make possible a more reliable communication [9]. Other approaches appeared from specific vertical applications such as in [10], where cattle monitoring is considered, where “ADR techniques to most efficiently find the optimal data rate for a firmware update” can be found. The importance of ADR is highlighted in [11], where a “study of environmental parameters impact in LoRaWAN” is carried out, concluding that “snowing leads to high fluctuations in SNR and RSS when Adaptive Data Rate (ADR) is disabled”. Finally, recently in [12], the authors extend their previous work [8] applying a variable-order hidden Markov model to predict the ED mobility pattern in the case of “unknown or undefined trajectories”. Eventually, the recent paper [13] in Table 1 presents a survey of different ADR algorithms that appeared in the literature.

It must be highlighted that it is possible to achieve a quite precise location of the ED in the LoRaWAN networks, just relying on the LoRa signal received from the gateways. For example, in [14] the authors report the results of two measurements’ campaigns by using a machine learning approach and conclude that “LoRaWAN-based localization with relatively dense gateways (GWs) deployment allows for achieving a meter-level accuracy, which may be suitable for the localization of workers”. Furthermore, in [15] it is reported that the LoRa Cloud ^™^, a tool from Semtech providing “geolocation services based on TOA and/or RSSI observations”, has been made available worldwide. However, the location capability is still not widely implemented in LoRaWAN networks, due to its technical complexity and, more importantly, the cost related to its implementation, especially on a large scale.

The main contribution of our paper is to propose a technique, based on machine learning, to classify the level of mobility of an ED relying purely on the data available on the NS, without the need to resort precise location techniques, based on the processing of physical signals, e.g., by making use of the time of arrival (ToA) used in [15]. Based on this classification, a tuning of the ADR can be made reliably, having, for example, different possible ADR algorithms for different levels of mobility. As a matter of fact, what is actually important for optimizing the ADR is not the specific trajectory a node is taking but the mobility level, i.e., if it is fixed or mobile. Furthermore, specific optimizations can be performed in the ADR in the case of ED which are in “deep indoor” installations, such as energy or water meters. These nodes transmit their packets infrequently, but good reliability is needed so they need an ADR algorithm privileging the reliability of the data rate. We would like to remark that our work is quite peculiar and specific and—to our knowledge—it is the first of its kind. As a matter of fact, we are not aiming at localizing the LoRaWAN node, a problem addressed by many papers such as the recent one [16]. Our aim is to detect the level of mobility. Of course, the level of mobility can be derived from the positions of the node in subsequent time instants, but our method is much simpler and does not require precise localization, working only on the data present at the network server.

The remainder of the paper is organized as follows. Section 2 gives an overview of the involved technologies. Section 3 introduces the machine learning algorithm to classify the node. In Section 4, we present the experimental environment. Finally, in Section 5 we analyze the results from our experiments and we draw the conclusions.

## 2. An Overview on LoRa and LoRaWAN

A LoRaWAN network is based on two protocols: LoRa, which regulates the physical layer communication between the EDs, and LoRaWAN, the medium access control protocol used on top of LoRa. In the following section, we will investigate more deeply the protocols.

### 2.1. LoRa

LoRa is a proprietary physical layer technology patented by Semtech [17], based on chirp spread spectrum (CSS) modulation techniques that enable long-distance and low-power communications. It operates in the sub-GHz ISM band, and it is an M-ary digital modulation [18,19], whose waveforms are not perfectly orthogonal [20]. Each LoRa transmission is characterized by four parameters: spreading factor (SF), transmission power, bandwidth, and coding rate. All these variables are managed and controlled by an adaptive data rate mechanism, which is part of the MAC protocol.

### 2.2. LoRaWAN

In contrast to the proprietary PHY layer LoRa, the remaining part of the stack protocol, known as LoRaWAN, is open, and it is developed and maintained by the LoRa Alliance. The LoRaWAN network is typically deployed in a star-of-stars topology, where the EDs are connected through a single-hop link to one or many gateways, which, in turn, forward the packet traffic toward a common network server via standard IP protocols. The gateways act as a simple bridge between the end nodes and the network server; in fact, after decoding and adding some information regarding the quality of reception to the packets, they forward every message to their network server.

The MAC of the LoRaWAN networks is essentially an ALOHA protocol controlled by the network server, which is in charge of assigning the transmission parameters to the end node by means of an adaptive data rate (ADR) mechanism.

It must be highlighted that although the architecture of LoRaWAN resembles that of a cellular network, the LoRaWAN networks are “cell-free”. An ED is not belonging to any “cell” identified by a GW, and there is no such a concept as “handover”. Any packet sent by an ED is picked up by any GW that is able to decode it and all of the GW decoding the packet are sending it to the NS, which deduplicates the packets, selecting the one received with the best quality.

A final remark on this Section is in order: the LoRa modulation and the LoRaWAN system are quite active areas of research and development, in academia and the industry. We would like to mention the paper [21] for a recent survey and the paper [22], which testifies to the increasing interest for LoRa modulation and LoRaWAN system for satellite communications in low Earth orbit, something hardly predictable a few years ago.

## 3. Machine Learning Algorithm

The aim of this work is to find a technique to classify the EDs in a LoRaWAN network into the following three categories: fixed, mobile, and deep indoor. The main idea is to use a machine learning approach and find a function that is able to predict the category of the EDs, starting from the information collected by the received packets at the GWs. We suppose to have a very small set of EDs that are already labeled, whose reception data, collected at the gateway, can be used as the training set for our algorithm. This set will be our ground truth. It is important to clarify that the resultant function of the algorithm for the categorization of nodes will be specific to the LoRaWAN deployment where it was computed.

To reach our goal, we will use a supervised learning algorithm, the support vector machine (SVM). SVM is a very useful machine learning tool for learning linear predictors in high-dimensional feature spaces. The main idea behind SVM is to find the best boundary (or hyperplane) that separates the different classes in a dataset by maximizing the margin, which is the distance between the hyperplane and the closest data points from each class. These points are the so-called support vectors; in fact, because they are the closest points to the decision boundary, they have the most impact on the position of the hyperplane. SVM is particularly useful when the data have many features, or when the classes are highly nonlinear. The algorithm’s ability to handle nonlinearly separable data is obtained with the kernel method technique, which transforms the data into a higher-dimensional space where the data points become linearly separable. This enables SVM to model complex, nonlinear decision boundaries that cannot be classified by other linear methods. Furthermore, SVM is a robust algorithm that is not affected by the presence of noise or outliers in the data. With the regularization parameter *C* it can be possible to balance the tradeoff between maximizing the margin and minimizing the classification error. In this paper, we use the soft version of the SVM and the kernel method, which enables us to enrich the expressive power of halfspaces by first mapping the data into a high-dimensional feature space, and then learning a linear predictor in that space [23]. The proposed algorithm running on a computer with a ninth generation i7 CPU and an RTX 2060 GPU takes around one minute for the training phase, and it is almost immediate for the testing phase.

The creation of the features to represent the nodes is done by collecting the reception information of the packets from every device; in particular, we will use: the packet received signal strength indication (RSSI), the packet signal-to-noise ratio (SNR), the number of gateways receiving the packet from the same ED and the number of packet transmissions for every successful packet reception. After the reception of some packets from the same ED we can compute the node features described in Table 1. We remark that we do not use all these features in the table to represent a node, but select only the ones that are most representative for the EDs of the specific LoRaWAN network. In fact, the more feature we use, the bigger the dimensionality of the problem becomes, with the risk of overfitting the dataset. The main reason behind this is that the employed dataset is too small for the use of a too-high dimensional feature space. So from repeated tests, we have found that the most representative features are: std RSSI, std SNR, mean RSSI and mean SNR.

After selecting the features, the training set of EDs (typically around a few hundreds of EDs) to get the classification function takes place. By using the training set we can select the minimum number of packets before computing the node features; it is clear that the more packets we use the more precise will be the classification, but this comes at the cost of a slower procedure to profile the EDs. Consequently, there is a tradeoff between speed and precision. At the same time, the larger the set of EDs used to train the algorithm the more accurate will be the final classification.

In the following section, we will apply this method to a real LoRaWAN deployment [24].

## 4. Experimental Setup

In this section, we apply the method described above to a real LoRaWAN network deployment, whose node distribution is reported in Table 2. In particular, we started our analysis from a dataset containing all the packets sent in the network during one month. Then we grouped the packets with respect to the transmitting ED, and we kept only the EDs that have sent more than 20 packets. At this point, we were able to compute the features of each device.

These decisions lead us to a dataset composed of 1763 EDs, or samples, distributed between the three classes as shown in Table 3. As we can see from the Table 3, our dataset contains too few EDs in the class “deep indoor”, so in the remainder, we focus our considerations on the classes “mobile” and “fixed”. We expect similar results for the “deep indoor" class, should we have had a dataset including more EDs belonging to this class.

**Figure 1:**
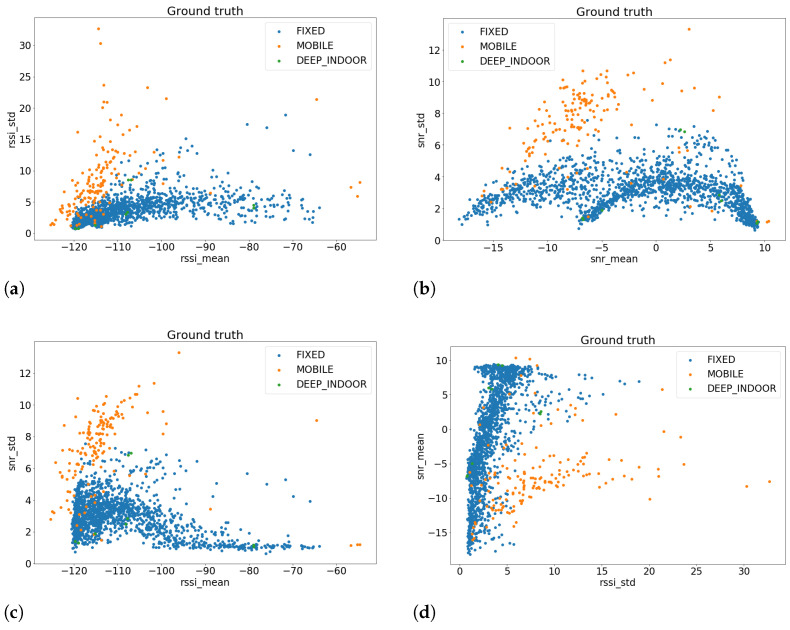
Plots of the most representative features of the EDs in the LoRaWAN network: (**a**) std RSSI, (**b**) std SNR, (**c**) mean RSSI, and (**d**) mean SNR.

In Figure 1, we have plotted the most interesting feature combinations, where the three classes are more distinguishable. As we can notice, the most representative features are std RSSI, std SNR, mean RSSI, and mean SNR, which will be used as descriptors for our samples.

To enhance the flexibility of the soft SVM, we have also adopted the kernel method with the following kernel functions: linear kernel, polynomial kernel, and radial basis function kernel, which are usually used to introduce our prior knowledge in the learning algorithm for the classification problem. To select the best model to represent our samples we will use a validation set, which is a part of the dataset not used to train the algorithm. In our specific case, because the data is scarce and we do not want to “waste” precious samples on validation, we will use k-fold cross-validation. In k-fold cross validation, the original training set is partitioned into *k* subsets (or folds) of size m/k. For each fold, the algorithm is trained on the union of the other folds and then the error of its output is estimated by using the fold. The average of these errors is the estimate of the validation error, which will be used to determine which is the best model. Then the algorithm is retrained using this model on the entire training set.

To develop the SVM tool in Python we have used the package scikit-learn [24], which has implemented the SVM in the class sklearn.svm.SVC.

Before the real part of machine learning, we performed a normalization process with the aim to normalize all the data inside the interval [0,1], which is a fundamental step for the SVM techniques. To find out the best model to perform the predictions on our dataset we use the class sklearn.model_selection.GridSearchCV, which implements the SVM for all the possible parameter combinations in Table 4 and by means of k-fold cross-validation finds the best model for our data.

The parameters required by the SVM implemented in scikit-learn are as follows.

- C, which controls the precision in the classification. In other words, a high value of C aims at correctly classifying all the samples during the training phase, whereas a smaller value aims at a softer classification. It is related to the weights associated with the slack variables that we have introduced in the previous section.
- Kernel, which is the transformation applied to the data samples before the SVM method. It can be linear (linear function), poly- (polynomial function), or rbf (radial basis function).
- Gamma, which tunes the shape of the kernel functions.
- Degree, which is the degree of the polynomial kernel. It is meaningful only if the kernel is equal to poly-.

As we have already anticipated, the classification is performed between the mobile and fixed EDs, because the class of deep indoor EDs has very few nodes.

**Figure 2:**
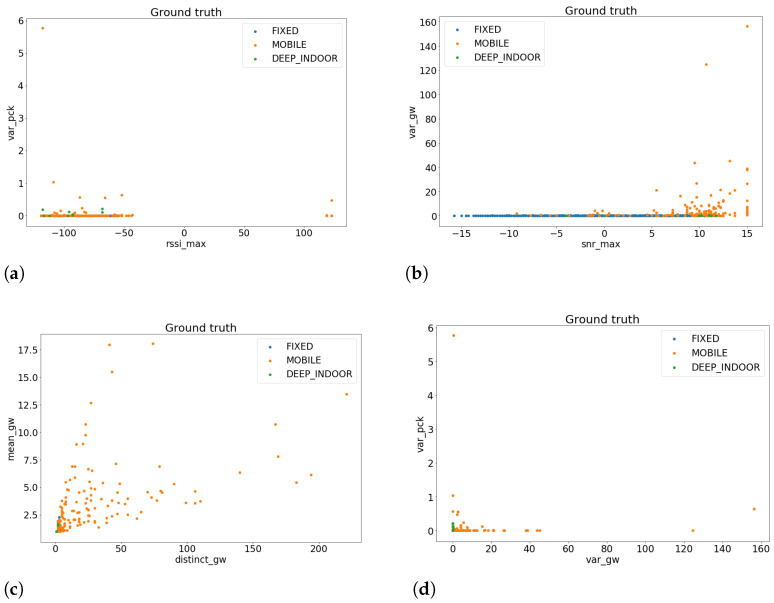
Plots of the worst samples’ features. (**a**) RSSI max vs. Var PCKs; see Table 1; (**b**) SNR max vs. Var GW; see Table 1; (**c**) Distinct GW vs. mean GW; see Table 1; (**d**) Var GW vs. Var PCKs; see Table 1.

We remark that, for the learning task, we will not use all the features that we have previously extracted, because the more feature we use the bigger the dimensionality of the problem, and then the higher the risk of overfitting the dataset. The reason is that the dataset is too small for the use of complex learning methods. Moreover, not all features are meaningful to extract some patterns from the data, as we can see from Figure 2.

**Figure 3:**
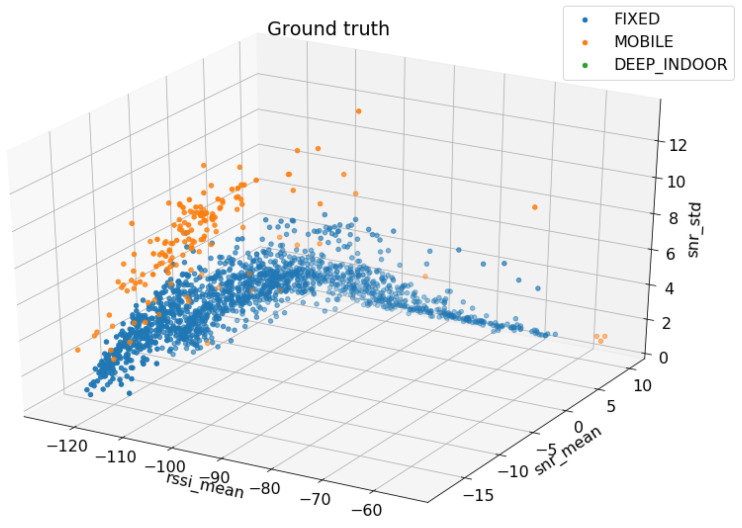
Example of 3D plot of the most relevant features.

From repeated tests, we have found that the most representative features for the examples are: mean RSSI, standard deviation of the RSSI, mean SNR, standard deviation of the SNR and mean number of packet transmissions per ED (Figure 3).

As a consequence, our samples will have a dimension d=5, which leads to a VC-dimension (see [23]) equal to d+1=6 for the hypothesis set of halfspaces. We remember that the VC-dimension is an important parameter for the learning algorithm, which, if equal to a finite number, guarantees the probably approximately correct (PAC) learnability of the hypothesis class (for more details on this topic please refer to [23]).

Because the total number of mobile EDs is small with respect to the number of fixed EDs, we have decided to train the SVM with a special dataset with 120 EDs from each of the two classes. The results are very good and we reach a validation error, equal to 98.05%, and a test error, calculated in the remaining part of the dataset not used for the training part, equal to 99.67%. The best model selected through the grid search is the one with a Gaussian kernel, γ=1 and C=100. To get a more precise insight into the precision of the algorithm, we have also created a confusion matrix, which is a specific table layout that allows the visualization of the performance of an algorithm. Each column of the matrix represents the instances in a predicted class whereas each row represents the instances in an actual class. The name stems from the fact that it makes it easy to see if the system is confusing two classes. From the confusion matrices in Table 5 and Table 6, we can see that the algorithm is quite precise in classifying the nodes, even though it sometimes tends to misclassify some nodes.

## 5. Conclusions

In conclusion, the proposed SVM algorithms can learn a very good model to solve our classification problem, i.e., determining if an ED is mobile or fixed, even though the dataset is not so rich. Our algorithm, validated in with a real-world dataset, can then be a solid foundation for selecting the best ADR algorithm depending on the mobility of an ED.

## Figures and Tables

**Table 1:** Features computed to classify the end nodes in the LoRaWAN network.

Node Feature	Description
Max RSSI	maximum RSSI among all the packets transmitted by the ED
Min RSSI	minimum RSSI among all the packets transmitted by the ED
Mean RSSI	mean RSSI for all the packets transmitted by the ED
Std RSSI	standard deviation of the RSSI for all the packets transmitted by the device
Max SNR	maximum SNR among all the packets transmitted by the ED
Min SNR	minimum SNR among all the packets transmitted by the ED
Mean SNR	mean SNR for all the packets transmitted by the ED
Std SNR	standard deviation of the SNR for all the packets transmitted by the device
Distinct GWs	number of distinct gateways that have received at least one packet from the device
Mean GWs	mean number of distinct gateways that receive each packet from the device
Var GWs	variance of the number of distinct gateways that receive each packet from the device
Mean PCKs	mean number of packet transmissions for every packet from the device
Var PCKs	variance of the number of packet transmissions for every packet from the device
Max TXs	maximum among all the total number of transmissions needed to correctly deliver every packet by the device

**Table 2:** Node types inside the LoRaWAN network.

Class	Type	Number
*fixed*	waste bin	1506
	parking meter	16
	ground humidity sensor	14
*mobile*	tracker	221
*deep indoor*	water meter	29
	environmental sensor	7

**Table 3:** Distribution of the filtered EDs between the three classes.

Class	EDs	Percentage
*mobile*	153	8.68%
*fixed*	1601	90.81%
*deep indoor*	9	0.51%

**Table 4:** Parameters used for the grid search of the SVM.

Parameter	Values
C	$\left\{10^{-2},10^{-1},1,10,10^{2},10^{3},10^{4},10^{5}\right\}$
kernel	linear, poly, rbf
gamma	{0.1,1}
degree	{2,3,4}

**Table 5:** Confusion matrix.

	Predicted Class		
**Actual Class**		Fixed	Mobile
	Fixed	1479	2
	Mobile	4	29

**Table 6:** Confusion matrix normalized with respect to the predicted class.

	Predicted Class		
**Actual Class**		Fixed	Mobile
	Fixed	1.00	0.06
	Mobile	0.00	0.94
